# Prevalence and pattern of domestic accidents in the field practice area of Jawaharlal Institute of Urban Health Centre, Puducherry: a cross-sectional analytical study

**DOI:** 10.5249/jivr.v12i1.1182

**Published:** 2020-01

**Authors:** Tanveer Rehman, Sudheera Sulgante, Sitanshu Kar Sekhar

**Affiliations:** ^*a*^Department of Preventive and Social Medicine, Jawaharlal Institute of Postgraduate Medical Education and Research (JIPMER), Puducherry - 605008, India.

**Keywords:** Domestic accidents, Household hazards, Safety practices, Urban health

## Abstract

**Background:**

Injuries constitute around 16% of the total disease burden in India with respect to Disability-Adjusted Life Years. More than two third of these injuries are unintentional and occur at household level. Domestic accidents are preventive and can be drastically reduced by effective measures and safety consciousness. This study aims to find the prevalence of domestic accidents, the household safety practices and their association with socio demographic factors in selected urban wards of Puducherry.

**Methods:**

A population-based cross sectional analytical study was conducted in the service area of Jawaharlal Institute of Postgraduate Medical Education and Research Urban Health Centre, Puducherry, in June 2018. Data regarding self-reported domestic accidents in the last one year were collected using a structured questionnaire and assessment of household hazards was done after examining the houses.

**Results:**

Among the 578 randomly selected households, 393(68%) belonged to nuclear families, 486(84%) had pucca houses and in 339(59%) overcrowding was present. 59(10.2%) households reported domestic accidents – of which 25(42%) had adult victims, 22(37%) were due to falls, 27(45%) had upper limb injuries and 25(43%) occurred in kitchen. On assessment of safety practices, 121(21%) houses had doors with stoppers, 394(68%) had items scattered on living room floor while 128(24%) and 160(30%) had grab bars and doormat in bathrooms respectively. Domestic accidents were more prevalent among overcrowded households - Prevalence Ratio: 1.74 ([95% CI: 1.02 – 2.98], p = 0.04).

**Conclusions:**

The prevalence of domestic accidents was 10.2% in the present study. It was reported mostly among the adults and in the kitchen, with falls being the most common cause and upper limbs injury being commonest. Most of the houses had objects lying scattered on the floor hindering movement; and stoppers and grab bars were missing from the doors and bathrooms respectively. Overcrowding was significantly associated with domestic accidents.

## Introduction

Injuries constitute around 16% of the total disease burden in India with respect to Disability-Adjusted Life Years.^1^ More than two third of the injuries are unintentional and more often at the household level. ^2^ In many developed nations more accidents happen in home than anywhere else. ^3^ The statistics is worse in developing nations like India, especially in rural areas and urban unplanned towns. ^4,5^ Since these are always unexpected and unplanned, we do not routinely think about injury risk during our day-to-day interactions, domestic accidents being one of them. 

Domestic accidents are the accidents which happen in house or in its immediate surroundings and not associated with traffic, vehicles or sport.^6^ The most common ones being drowning, burns, falls, poisoning and injuries from sharp, pointed objects or animals. In 2015, drowning contributed to 7.2% of all the accidental deaths, whereas accidental poisoning, fire and falls led to 6.3%, 4.3% and 4.1% of all accidental deaths respectively in India. ^7^ Every year more than five lakh individuals get injured due to domestic accidents. ^8^ This leads to physical disabilities, permanent deformities, psychological problems, premature death, economic burden and grief - not only to the victim but also to the entire family. 

Domestic accidents depend on the physical and social environment and also on the functional capacity of the individual. Different age groups have disparate factors like the elderly age group have low vision, slow reflexes while the toddlers do not achieve the cognitive maturity and coordination to predict the hazardous steps when not under supervision.^9,10^ It is generally accepted that domestic accidents are preventive and can be drastically reduced by effective measures and safety consciousness, e.g. preventing poor unplanned house constructions in urban slums. ^11^ Most of the domestic injuries are considered to be minor injuries and do not reach the level of health care intervention. ^12^ Whereas, on the other hand, hospital- based studies focus only on the cases which necessitate medical attention and so will not reflect the nature of domestic accidents occurring in the community. ^13^ So facility-based studies do not represent the true burden of domestic injuries occurring in the community. Hence there is a paucity of knowledge regarding domestic accidents and its awareness. ^14^ To address this gap, we conducted a house-to house community-based study to find burden of injuries occurring at the household level in our field practice area. Moreover, this information would aid in conducting health education sessions at the community level and launch a comprehensive injury prevention programme in our service areas. Thus, this study aims to find the prevalence of domestic accidents, the household safety practices and their association with socio-demographic factors in selected urban wards of Puducherry. 

## Methods

**Study design and setting:** A population-based cross sectional analytical study was conducted in the service area of Jawaharlal Institute of Postgraduate Medical Education and Research (JIPMER) Urban Health Centre (JIUHC), Puducherry in June 2018. JIUHC provides general outpatient services through medical officer, senior and junior residents, and interns for six days in a week to a population of about 9423 with 2501 households spread over four wards along the coastal areas of Puducherry. All the four wards are similar in socio-demographic and economic characteristics.

**Study participants:** Adults (18 years and above), who were the decision makers or who were present in the house during the interview time were the respondents. If any house had more than one participant qualified to be a respondent, then one was selected by lottery method. 

**Sampling population:** All residents permanently residing in the four wards were eligible to participate in the study.

**Sample size:** Sample size was calculated using OpenEpi (v 3.01 updated on 2013, USA). ^15^ Based on a previous community-based study, ^16^ assuming proportion of domestic accidents as 0.15, relative precision of 20% and alpha error of 5%, the minimum sample size obtained was 544 households and considering a non-response rate of 10%, the sample size required was estimated to be 600 households.

**Sampling technique:** Number of households was selected from each urban ward based on proportionate to size. A line list of 2501 households with their address details maintained by JIUHC center staff was used for selection of households. Using systematic random sampling method, every fourth household was approached. Right hand rule was used for selection of houses.

**Study procedure:** Ten MBBS interns posted in JIUHC from the Department of Preventive and Social Medicine, JIPMER, were chosen as data collectors. They were sensitized regarding the objectives of the study, confidentiality of information, participants’ rights, informed consent and were also trained to administer the questionnaire to the subjects. After obtaining the service area map and total number of households from each urban ward, the number of households to be selected from each of the four urban wards was decided and based on that the interns were divided. A random number was chosen between one and four before starting of data collection from each urban ward and after that every fourth household was selected till the desired number was achieved in each ward. Then from the selected household after obtaining informed verbal consent from the eligible participant, questionnaire was administered at their own residence. Two resident doctors from the same department posted in the center supervised the data collection procedure by reviewing all the questionnaires at the end of each day to ensure completion of data collection forms and addressed any issues faced by the interviewers. Data were collected house-to-house and if any house was locked or respondent was busy with household chores or refused to participate, then the next house was approached. The participants were contacted only once for the purpose of the study and data collection happened five times in a week for a total study period duration of two months.

**Study tool:** Interview was conducted using a structured questionnaire which had three parts. First section comprised of the socio-demography, second part had details regarding self-reported domestic accidents^6^ in last one year and the last part consisted of a checklist of six components having 43 parameters for assessing the household hazards and safety practices followed by the family members after examining the house. Since no standard questionnaire was available to assess household hazards and safety practices, we framed the questions based on other studies.^1,4,5,11-13^ The questionnaire was originally developed in English. It was translated into the vernacular language (Tamil) and was cross checked by back translating to English by two bi-linguistic experts who were well versed in English and Tamil. It was pretested in 30 households and modified accordingly. Face and content validity were ensured by assessment of the items by the authors and exhaustive literature review.^17^ An internal consistency test was performed on the 43 parameters using Cronbach’s alpha value. The scale reliability coefficient value was 0.8 which is considered highly reliable. ^18^ At the end of the interview, participants were briefed and counselled regarding the hazards present in their respective homes. 

**Statistical analysis:** Data were entered using Epicollect5 (Developed by Imperial College, London)^19^ and analyzed using STATA version 14.0. Continuous variables like age of victim and household hazard scores were summarized as mean (standard deviation) and median (interquartile range) respectively based on distribution of the data. Categorical variables like the type of house, religion and educational status were summarized as frequency (percentage). All the 43 parameters having dichotomous outcomes were allotted binary scores (0 &1) based on the hazards present in the house. For example (Table 3), in the first component ‘entry/exit of the house’: houses with ‘doors provided with stopper’ were given score zero (as it is a safe or good practice) while those without it was given score one (as the door can suddenly close causing injury); similarly houses with ‘lighting’ were given score zero while without it score one (as more prone to injury in dim vision). So, the hazard scores for all the 43 items in a single house were added and finally every house had a household hazard score. Higher the household hazard score, more prone or riskier was the household to domestic accidents. Using the interquartile range, the households were categorized into Low Risk (0-10 score), Moderate Risk (11-17 score) and High Risk (>17 score) to find the association with domestic accidents. Prevalence of domestic accidents was summarized as proportion with 95% confidence interval (CI). Bivariate analysis (Chi square test/Fisher exact test) was used to find the association between socio-demographic factors and domestic accident. The strength of association of domestic accidents with independent categorical variables was expressed using prevalence ratio (PR) with 95% CI and was calculated by logistic regression. A p value ≤ 0.05 was considered as statistically significant.

## Results

A total of 600 households were contacted - out of which 578 were included in the study. Rest 22 individuals were not able to understand and respond to the complete questionnaire making the response rate as 96%. 

Table 1 depicts the socio-demographic characteristics of the study households. Of total, 68% (n=393) belonged to nuclear families, 84% (n=486) had pucca houses, 56% (n=326) had Below Poverty Line (BPL) cards and 59% (n=339) had overcrowded households. Among the head of the families, 30% (n=173) studied till secondary school followed by 21% (n=120) till middle school.

**Table 1:** Socio-demographic characteristics of the study households residing in selected urban wards of Puducherry, 2018 (N = 578).

Characteristics	Number	(%)*
**Type of family**		
Nuclear	393	(68)
Joint	98	(17)
Three generation	87	(15)
**Religion**		
Hinduism	472	(82)
Christianity	88	(15)
Islam	18	(3)
**Educational status of Head of Family**		
Illiterate	63	(11)
Primary (till 5th standard)	55	(9)
Middle (till 8th standard)	120	(21)
Secondary (till 10th standard)	173	(30)
Higher secondary (till 12th standard)	79	(14)
Graduate (College and above)	88	(15)
**Ration card**		
BPL (Below Poverty Line)	326	(56)
APL (Above Poverty Line)	242	(42)
None	10	(2)
**Type of house**		
Pucca	486	(84)
Semi – pucca	86	(15)
Kutcha	6	(1)
**Overcrowding**		
Yes	339	(59)
No	239	(41)

*Column percentage

The prevalence of domestic accidents was 10.2% [95% CI: 7.9 – 13] among 578 households in JIUHC service area of Puducherry in June 2018.

Table 2 shows the details regarding the 59 domestic accidents reported in the last one year. There is almost equal gender distribution among the reported accidents. The median age of the victims was 27 (10 - 52) years. Upper limb (46%, n=27) was the most common location of bodily injury while there were no abdomen or neck injuries reported. The accidents mostly took place during the morning hours (36%, n=21) in the kitchen and predominantly were due to falls (37%, n=22) and injuries with sharps (31%, n=18). For treatment, study participants either went to government facility (37%, n=22) or treated themselves at home (36%, n=21). 

**Table 2:** Details regarding the domestic accidents in last one year in the study households residing in selected urban wards of Puducherry, 2018 (N = 59).

Characteristics	Number	(%)*
**Gender**		
Males	29	(49)
Females	30	(51)
**Age group (in years)**		
Child (0-10)	15	(25)
Adolescent (11-17)	8	(14)
Adult (18-59)	25	(42)
Elderly (≥ 60)	11	(19)
**Type of injury**		
Fall	22	(37)
Injury by sharp instruments	18	(31)
Burns	9	(15)
Accidental poisoning	9	(15)
Choking	1	(2)
**Bodily location**		
Head	14	(24)
Chest	3	(5)
Upper limbs	27	(46)
Lower limbs	15	(25)
**Place of injury**		
Entrance/exit	4	(7)
Living room	17	(29)
Kitchen	25	(43)
Bathroom	3	(5)
Staircase	2	(3)
Bedroom	3	(5)
Courtyard/veranda	5	(8)
**Time of injury**		
Morning	21	(36)
Afternoon	11	(19)
Evening	15	(25)
Night	12	(20)
**Health seeking behavior**		
No treatment	12	(20)
Home treatment	21	(36)
Government facility	22	(37)
Private facility	4	(7)

*Column percentage

Table 3 denotes assessment of safety practices followed by the 578 study households. Only one out of every five houses had doors and windows with stoppers (21%, n=121 and 20%, n=85 respectively). At the entrance or exit, height of the door was not too low in 76% (n=440) and floor was not slippery or prone to accident in 70% (n=403) of the houses. Rugs or objects were lying scattered on floors in 68% (n=394) of living rooms and 66% (n=273) of bedrooms. In the living room, most of the houses had windows (73%, n=422) and did not have furniture with sharp edges (69%, n=399) or that would collapse (73%, n=423). Of total 458 (79%) homes having dustbins, 36% (n=164) covered their dustbins. Majority had separate the kitchen in their homes (83%, n=481) and kept inflammable items one arm away from gas stove (75%, n=434), matchboxes (72%, n=416) and pots handles (65%, n=378) above the shelf or in close cabinets to keep out of child’s reach. Among the 541 (94%) houses having bathroom, 24% (n=128) and 30% (n=160) had grab bar and doormat respectively for their bathrooms. More than half of the study population (57%, n=233) had electrical appliances left plugged in the sockets in their bedrooms. Majority of the households had lighting facility present in all the rooms and entry/exit of the house (Table 3). On allotting the scores as described above, the median household hazard score was 12(10 - 17). 177(31%), 278(48%) and 123(21%) households belonged to Low, Moderate and High-Risk categories respectively.

**Table 3:** Assessment of safety practices followed by the study households residing in selected urban wards of Puducherry, 2018 (N = 578).

Characteristics	Number	(%)*
**I. Entrance/ Exit of house**		
1.Doors provided with stopper	121	(21)
2.Lighting	478	(83)
3.Height of the door NOT too low	440	(76)
4.NOT slippery/steep/cracked/uneven	403	(70)
**II. Living room**		
5.Windows present	422	(73)
6.Windows with railing^†^	297	(70)
7.Windows with stopper^†^	85	(20)
8.Stable furniture	423	(73)
9.Lighting adequate	488	(84)
10.NO rugs/objects lying on floor	184	(32)
11.NO sharp edges	399	(69)
12.Wires NOT lying on the floor	421	(73)
13.Fan NOT within reach of children	401	(69)
14.NO medicine within child’s reach	407	(70)
**III. Kitchen**		
15.Separate kitchen	481	(83)
16.Inflammables kept away from stove	434	(75)
17.Lighter/matchbox out of child’s reach	416	(72)
18.Sharp items in a separate cabinet	348	(60)
19.Dustbin present	458	(79)
20.Dustbin closed^‡^	164	(36)
21.Smoke vent/windows present	301	(52)
22.Lighting adequate	494	(86)
23.Pots handles out of child’s reach	378	(65)
24.Kerosene NOT kept in regular bottles	375	(65)
25.Stool/chair NOT used to reach things	307	(53)
26.Floor NOT slippery	475	(82)
**IV. 27. Bathroom^§^**		
28.Door lock/latch present	484	(90)
29.Grab bar	128	(24)
30.Lighting adequate	472	(87)
31.Doormat outside	160	(30)
32.Water does NOT spill outside	353	(65)
33.Slippery floor NOT present	387	(72)
**V. 34. Staircase^ll^**		
35.Railing present	196	(76)
36.Steps stable/NOT slippery	193	(75)
37.Sharp edged steps NOT present	196	(76)
38.Items NOT blocking way	187	(73)
**VI. 39. Bedroom^¶^**		
40.Adequate lighting	365	(89)
41.Electrical appliances NOT left plugged in	179	(43)
42.Objects NOT scattered around	139	(34)
43.NO Sharp edges	344	(84)

*Column percentage; †422 houses had windows; ‡458 houses had dustbin; §541 houses had bathroom; ll 257 houses had staircase; ¶ 412 houses had bedroom.

Table 4 shows association of socio demographic characteristics with domestic accidents. Domestic accidents were 1.74 times more prevalent (PR 1.74 [95%CI: 1.02 – 2.98]) among overcrowded households and the finding was statistically significant (p value –0.04). 

**Table 4:** Association of socio demographic characteristics with domestic accidents among the study households residing in selected urban wards of Puducherry, 2018 (N = 578).

Characteristics	Total	Accidents, n (%) *	PR	95%CI	p value
**Total**	**578**	**59 (10)**			
**Type of family**					
Nuclear	393	35 (9)	1	-	-
Joint / Three generation	185	24 (13)	1.46	0.89 – 2.38	0.13
**Educational status of family head **					
Illiterate	63	6 (10)	1.68	0.54 – 5.25	0.38
Primary (till 5th standard)	55	5 (9)	1.6	0.49 – 5.27	0.44
Middle (till 8th standard)	120	20 (17)	2.9	1.08 – 7.18	0.03
Secondary (till 10th standard)	173	17 (10)	1.73	0.70 – 4.77	0.27
Higher secondary (till 12th standard)	79	6 (8)	1.34	0.42 – 4.21	0.62
Graduate (College and above)	88	5 (6)	1		
**Ration card**					
BPL (Below Poverty Line)	326	40 (12)	1.63	0.97 – 2.74	0.07
APL (Above Poverty Line)/ none	252	19 (8)	1		
**Type of house**					
Pucca	486	47 (10)	1		
Semi – pucca/ Kutcha	92	12 (13)	1.35	0.75 – 2.44	0.32
**Overcrowding**					
Yes	339	42 (12)	1.74	1.02 – 2.98	**0.04**
No	239	17 (7)	1		
**Risk of accident**					
Low	177	13 (7)	1		
Moderate	278	36 (13)	1.77	0.96 – 3.23	0.07
High	123	10 (8)	1.11	0.5 – 2.44	0.8

PR – Prevalence Ratio; CI: Confidence Interval; *row percentage

## Discussion

The prevalence of domestic accidents at the household level in an urban population of Southern India was found to be 10.2%. Domestic accidents were present in both genders almost equally and in the age group of 18 to 59 years. The most common type of injury was falls, injuring mostly the upper limbs and took place predominantly during the morning hours in the kitchen. Most of the houses had objects lying scattered on the floor hindering movement did not have their dustbins covered; and stoppers and grab bars were missing from the doors and bathrooms respectively. Every alternate house was prone to accident and overcrowding was significantly associated with domestic accidents. 

The prevalence of domestic accidents was 10.2% [95% CI: 7.9 – 13] among 578 households in JIUHC service area of Puducherry in June 2018. Other studies in urban and semi-urban areas showed a wide prevalence from 1.7% to 12.7%.^4,5,20,21^ Similarly, in rural population the range was from 4.8% - 14.6%. ^1,16,22-24^ The higher values in rural population could be attributed to the dearth of knowledge regarding household safety measures whereas the present study had a prevalence in the higher side because of the inclusion period of reported accidents in past one year compared to three and six months in other studies. Also, around half of the houses belonged to moderate risk category which could have contributed to the higher prevalence of domestic accidents in our study. Low socio-economic status, presence of overcrowding and single room houses could have also influenced the increased number of accidents. 

Domestic accidents were present in both genders almost equally. Studies by Krishna et al., Bhanderi and Choudhary, George et al. and Radhakrishnan and Nayeem however have shown females to be more affected while Netra et al, Kumarasamy and Prabhakar and Shawon studies have shown higher prevalence in males.^1,4,5,16,22-24^ There is a need for more studies to prove the probable gender association with domestic accidents. Most of the accidents were reported in the age group of 18 to 59 years – similar to studies by Krishna et al and Shawon.^16,23^ One out of every four houses that reported a domestic accident had a child victim. Supervision by the guardians can drastically reduce the risk of domestic accidents among the children. The most common type of injury was falls, injuring mostly the upper limbs, followed by injury by sharp instruments and this finding was supported by all the above studies. Majority of the accidents occurred in the morning and usually in kitchen in contrast to other studies where it occurred in the afternoon and courtyard. A probable cause for this difference could be that most of the houses included in our study did not have a courtyard; whereas most of the chores and hastening activities were done before going to work or school, which led to higher chance of accidents taking place in the morning. Regarding health seeking behavior, proportion of the participants getting treatment at government health facility and at home was nearly comparable. The presence of JIUHC in close proximity to the households would have facilitated this particular health seeking behavior. Also, they were familiar with the elemental emergency care measures related to the prevalent domestic accidents.

The households had adequate and appropriate infrastructure with respect to safety measures like illumination, height of doors, sharp edges; and competent knowledge like keeping inflammables away from stove, using dustbins and not keeping the floors slippery. The common hazards identified include absence of stoppers in windows and doors which could lead to unforeseen trauma; and scattered objects lying around in floor which can contribute to increased prevalence of falls. Nearly half of them did not keep sharp objects in the cabinets and did not have smoke vents in the kitchen. Majority of them left the dustbins uncovered. Exposed waste can attract insects as well as animals to the house which is a significant health risk. Kerosene kept in regular bottle in one third houses can serve as an accidental poisoning hazard. Majority did not have grab bars attached to bathroom wall and door mat outside the bathroom. The presence of grab bars can reduce the risk of falls in the bathroom whereas the absence of mat can lead to slippery floors and increase the risk of falls. Around half of the population kept the electric appliances plugged into the socket. This increases the risk of electrocution and occurrence of electric fires.

Domestic accidents were more prevalent among joint- or three-generation families, lower level of educational status of the head of family, BPL card holders, semi pucca or kutcha house owners and houses with moderate or high-risk categories and overcrowding; however, these were not statistically significant except overcrowding. 

So, with the rise in reported incidents, there is a definite need for frequent health education and awareness campaigns through mass media regarding the importance of household safety measures and its practice among the general population. Healthcare works need to target and identify the houses where overcrowding is present. Increased alertness and knowledge to be imparted to them on how to decrease the risk of accidents like objects should not be lying scatted on the floor, proper waste disposal and incorporating stoppers and grab bars. Further economic evaluation studies are required to decide on the frequency of screening of houses for domestic hazards and implementing this in national programmes. There is a need for larger cohort studies exploring the temporal association and consistency of the risk factors with domestic accidents. 

**Strengths and Limitations**

There are few strengths to this study. First, data collectors were well trained, supervision of the data collection and its validation was done. Second, the unobtrusive house examinations performed to identify the components of home-safety practices with such large sample size make the study unique. Probability sampling was done so the findings can be generalized to similar urban area settings in South India. The strength of association was expressed as PR; hence the over estimation, as with the case of odds ratio, was avoided. Finally, Epicollect5 software was used for data entry which would have reduced data entry errors. One of the major limitations of the study is the one-year long recall period bias in which many inconsequential injuries may have been missed. Home visit examination was a one-time observation by multiple data collectors, so cannot assume some of the practices like dustbin closure, loitering items on floor and plugging of appliances to be present always and there would have been interobserver variation regarding the same. Finally, in the analysis - the scores have not been given appropriate weighage even though the hazards may vary in severity and we did not calculate sample size to study associated factors of domestic accidents.

## Conclusion

The prevalence of domestic accidents was 10.2% which was significantly associated with overcrowding. Hence there is an absolute need for developing screening strategy to identify these houses and communicate household safety measures to the household members.

**Acknowledgements **

We extend our thanks to the study participants for support and the JIUHC staff and 2013 batch MBBS interns for their contribution in conducting this study.
